# Coloniality and the political economy of gender: Edgework in Juárez City

**DOI:** 10.1177/00420980211003842

**Published:** 2021-04-28

**Authors:** Jennie Gamlin

**Affiliations:** University College London, UK

**Keywords:** coloniality, masculinities, Mexico, territorial stigma, violence

## Abstract

The manner in which urban locations are drawn into the global economy defines their spatial organisation, distribution and utilisation. The relationships that are generated by this process include economic exchanges, racialised dynamics between workers and owners, gendered divisions of labour and the use and abuse of natural resources and infrastructure. These encounters of globalisation are often unequal or awkward and mediated by varying forms of violence, from structural to interpersonal, as these are used to rebalance the terms on which they meet. Using coloniality as an analytical tool, this article discusses the delicate balance of these Western-led encounters. Globalisation has become colonial by embedding hierarchical relationships in the foundations of the modern political economy. Gender identities, whiteness and non-whiteness, developed and underdeveloped are continually redefined, stigmatising certain groups and locations while elevating others on the basis of colonial power dynamics. Through a case study of the US–Mexico border city of Juárez, this article examines ethnographic work in its global context to explore how shame has become attached to male identities in locations of urban marginality. Theorising around the coloniality of urban space production, I discuss how Juárez’s border location has shaped its development though gendered and racialised frictions that are kept in check with violence. A coloniality perspective enables the unpicking of dominant conceptions of industrial cities in the Global South as metonyms for underdevelopment. Using the concept of edgework, I draw out how violence oils the wheels of globalisation to renegotiate damaged identities in contexts of territorial stigma.

## 摘要

城市地区融入全球经济的方式决定了它们的空间组织、分布和利用。这一过程产生的关系包括经济交流、工人和业主之间的种族主义动态、性别分工以及自然资源和基础设施的使用和滥用。这些全球化的遭遇往往是不平等或丑陋的，并以从结构性到人际性的各种形式的暴力为媒介，因为这些暴力被用来重新平衡全球化遭遇的条件。本文以殖民主义为分析工具，探讨了这些西方主导的遭遇的微妙平衡。通过将等级关系嵌入现代政治经济的基础，全球化已经变成了殖民主义。性别认同、白人和非白人、发达国家和不发达国家不断被重新定义，在殖民权力动态的基础上丑化某些群体和地方，同时抬高其他群体和地方。通过对美墨边境城市华雷斯城 (Juárez) 的个案研究，本文在全球背景下进行人类学研究，以探索在城市边缘地区男性身份是如何成为羞耻符号的。 围绕城市空间生产的殖民化理论，我探讨了华雷斯城的边境位置是如何通过性别化和种族化的摩擦影响其发展的，而这些摩擦又通过暴力被约束。从殖民主义的角度来看，可以将全球南方工业城市的主导概念解析为欠发达的转喻。利用边缘工作的概念，我刻画了暴力如何润滑全球化的车轮，在领土污名的背景下重新谈判受损的身份。

## Introduction: Juárez, the ‘urban Frankenstein’

Ciudad Juárez, a city of an estimated 1.5 million people, lies on the US–Mexico border directly south of El Paso, Texas, and its experience of urbanisation has been defined by this frontier location. The two cities, divided by the Rio Grande – now a concrete-lined waterbed – and an 18-foot high steel wall, have a combined population of 2.5 million, hundreds of thousands of whom cross this border in their daily lives. Children attend school on the ‘other side’, crossing on foot, while hundreds of millions of dollars pass through annually – much of this in the form of drugs and weapons. Ed Vulliamy, author of *Amexica: War Along the Borderline* (2010), talks of the beguiling lure of this ‘dusty and dangerous yet strong and charismatic city’ (Vulliamy, 2019). As Tsing (2005: 31) points out, frontiers are not natural or indigenous categories, but travelling theories or foreign forms, in this case a product of colonialism. Hence, ‘For years the adjoining cities have lived a symbiotic existence, their inhabitants, culture and economy an inseparable mix from either side of the increasingly militarised frontier’ (Eastaugh, 2017). Juxtaposed with the wealth of El Paso, Juaréz has been described as an ‘urban Frankenstein’ (Vulliamy, 2019), embodying the worst aspects and greatest contrasts of globalisation, that like all US–Mexico border zones is informed by the history and logic of crime and militarisation. Reflecting on her 2004 visit to the city, anthropologist Segato (2008: 79) describes how the city displays a ‘direct relationship between capital and death, unregulated accumulation and concentration and the sacrifice of poor, dark skinned, mestizo women’.

In this article, I will argue that the urbanisation of Juárez was defined by its coloniality with respect to the global economy and the specific gendered economic advantages that its physical location conferred. Using coloniality as an analytical tool, I will tell the story of its interconnectedness with urbanisation, revealing the functionality of violence as edgework, where violence is not a product of maldevelopment but part of the mechanics of globalisation though which hierarchies such as race and gender are negotiated. As the 2019 anti-Hispanic mass murder of 20 people in an El Paso Walmart exemplified (Romero et al., 2019), violence in its very many forms exists to protect the racial and gendered orders established under colonialism, on which this border economy was built.

The worlding of Juárez, the manner in which it is inserted into the global political economy, is as an industrial border city between the USA and Latin America, and the changing context of this borderline continually redefines patterns of gender, race and violence. Juárez became a border city almost by accident. The US–Mexico war ended in 1848 with the US annexation of over half of what was Mexican territory, and the deepest channel of the Rio Grande was chosen as the new frontier between the two nations. Although half of the river remained in Mexico, the US quickly began diverting the flow via irrigation channels to agricultural fields on the US side, ‘forcing the desert to bloom’ (Lobovits, 2018) on one side while drying up water sources south of the border (Stern, 2017). This infrastructural violence, also evident in the precarious housing of Juárez’s poorer neighbourhoods, may have been pivotal in the city’s later industrialisation and remains a defining characteristic of the unequal power relations between the US and Mexico. Through what Roy (2009) describes as transnationalism from below, Juárez was *worlded* by its burgeoning manufacturing sector which defined its location within the global economy on the basis of its position as the poor neighbour. Income opportunities, commerce and urbanisation itself happened in relation to this location beneath the USA. Here I use ‘beneath’ both geographically and symbolically as US imperialism advances itself as a role model for humanity, encapsulated by the idea of whiteness, the ‘ideal’ identity of a modern capitalist human (Echeverría, 2016). Hence, the physical contact between Juárez and El Paso is a constant reminder of this. On crossing the border between the US and Mexico, Stern (2017: 20) describes how cars coming into the United States scarcely seem to be moving, while they ‘breeze through into Mexico, no need for passports’. The differentiated status each country attributes to the other stigmatises Mexican lives, skin colours and neighbourhoods, devaluing goods and workers.

The 1990s heralded a new era of urbanisation, as NAFTA (the North American Free Trade Agreement) fomented the multiplication of *maquiladoras* (factories producing for export) along the border, increasing by 72% between 1993, when the agreement was signed, and 1997. This was mirrored by a 50% population increase in the decade to 2000 (Haley, 2010), as workers arrived from central and southern Mexico. Such expansion was facilitated by the city’s spatial distribution (Ballí, 2009). Located in the desert, Juárez is surrounded by expanses of privately-owned desert terrain, creating huge unpatrolled areas on the edge of the city. Between these spaces – which are increasingly populated with factories – and the city lie ‘large swathes of low-income residential neighbourhoods’, poor in communications and social and health infrastructure, that are ‘violently contested by associations of young men with few social and employment opportunities’ (Ballí, 2009: 15). Furthermore, the city’s layout is such that maquiladora workers must traverse great expanses of space between their homes and work, where there is often no safe public transport, making it a ‘dangerous spatial grid’ for women (Ballí, 2009: 16).

Femicides – murders where the specific motivation is the victim’s female gender – drew global attention to Juárez in the 1990s, when the compelling mixture of murder, maquiladoras and misogyny brought into focus extreme levels of gender violence. Anthropologist Melissa Wright describes how initial state/public security reactions pulled the concept of ‘public women’, a term that ordinarily refers to sex workers, into use for femicide victims. The notion of ‘public women’ diverges starkly from culturally dominant ideas of femininity that define a good woman as one who stays at home; in contrast, the concept of ‘public men’ equates with ‘citizen’ (Wright, 2011: 713; see also Orozco, 2019). Hence, femicide victims were blamed for their own fate, guilty of violating accepted gender norms. Such stereotypes are shaped by the specific coloniality of gender and power that has defined men and women in Juárez, throughout a modernisation process shaped by what Orozco (2019: 139) describes as the ‘masculine’ tourism and leisure industry that has commodified women’s bodies. The border location also gave Juárez unique advantages for illicit trade in goods, alcohol and drugs, defining a dark and gendered political economy as the weaker and cheaper sister of the two cities.

Femicide and homicide grew in parallel and by 2006 Juárez was the world’s murder capital, averaging between four and five homicides a day (Ballí, 2009: 175). International outcry led to increasing levels of militarisation, which in turn fuelled further violence, mainly in working-class neighbourhoods up against the border with El Paso. Criminals ‘tried to pass off as soldiers’ (Ballí, 2009: 182), with the latter representing a historical model for Mexican masculinity. Meshing together interpretations of gender, violence and public discourse, Wright (2011: 710) describes how violence became essential to the gendering of space, for ‘defining and controlling the modern liberal subject’ around the ‘exclusion of the feminine from the public sphere of politics, economics and culture’, with its subsequent impact on identity and culture. Gender violence reached its current magnitude thanks to the normalising impact of a culture of impunity, with Juárez gaining a reputation for lawlessness (Orozco, 2019). Murder was pitched as urban cleansing (Wright, 2011: 715) and fed though a channel of epistemic violence: shame and stigma defined these deaths as ‘stories that do not count’ (Davies, 2019: 1). Locations of death had become sites of territorial stigma, where poverty, gendered moral aberrations, criminality and delinquency fostered and justified a ‘politics of indifference’ (Davies, 2019: 13).

## Theoretical approach: Interconnectedness

Such characterisation as a city of death is inseparable from dominant concepts and expectations of urban modernisation and development, where ideas such as the ‘megacity’ have become a ‘metonym for underdevelopment’ (Roy, 2011). Here I disassemble this stereotype using as points of entry decolonial analyses that articulate gender, violence and urbanisation as interconnected historical processes.

There is transcultural evidence to support the notion that ‘masculinity is a status conditioned by its own obtention, that must be reconfirmed with certain regularity’ (Segato, 2008: 8; translation mine). Focusing on this need to reinforce masculinity, I will unpack the coloniality of gender as a process and consider how this occurred over time and place, in order to visibilise its role in the production of urban spaces. Edgework is the function of voluntary risk-taking in contexts of alienation. It has been extended by Lyng (1990) to explain the social context of gang violence, an analysis that mirrors other work on marginalised masculinities and is useful for understanding disruptions to the gender order (Bourgois, 2003; Hume, 2004). I extend the use of this concept to that of a functional strategy for redressing and negotiating territorial stigma, in this case that which is the product of ongoing colonial processes of urban identity formation.

As an analytical tool, coloniality considers colonialism as an ongoing and unfinished process, as opposed to a specific historical period. To this end, I argue that globalisation is the contemporary form of coloniality, as it structures relationships of race and gender in order to retain their usefulness to the global political economy. In so doing, I also bring a wide-angle lens to gender violence, contextualising it beyond the specific urban locality to describe its role in the worlding of Juárez, a historical process shaped by the coloniality of global power.

With Juárez City as a case study, I will examine the multilevel structural and historical processes that operate in the contexts of urban marginality to spoil identity, by bringing together published ethnographic research and critical theory to advance understandings of how coloniality has shaped urban spaces at their intersection with gender. I analyse research on gender violence in Mexico (Arteaga Botello and Valdés Figueroa, 2010; Azaola, 2009; González Montes, 2012) and ethnographies of violence and masculinity (Bourgois, 2003; Hume, 2004; Messerschmidt, 2000) to disassemble dominant discourse around violence in Juárez. The sources for the case study of Juárez are predominantly peer-reviewed ethnographic studies, where primary data have been generated and analysed to explore the concept of gender violence, and these have been supplemented with press articles for an up-to-date context.

## The coloniality of gender and violence

Historical and ethnographic analyses such as those of household, economy and kinship give indications of how gender was formed and transformed under colonialism, through post-colonialism, industrialisation and development. In this section, I describe how historical events and processes shaped the coloniality of gender and violence in Mexico. As an analytical tool, coloniality explains how the identities of coloniser and colonised were formed relationally – established in relation to each other. Gender as a social structure is also formed relationally, through interactions between people, and through the actions and expectations of people and groups that perform and respond to gendered behaviour. These roles are supported by a series of institutions and structures such as family, education systems and the labour market (Connell, 2005; West and Zimmerman, 1987). The Western sociocultural order, with gender at its centre, came to be seen as universal through colonialism and subsequent systems of coloniality (Mies, 1998; Quijano, 2000; Segato, 2010a). Hence, to borrow from Lugones’ (2008) term ‘the coloniality of gender’, non-Western gender systems were subsumed by colonial cultural and gender systems.

Globalisation is economic and social governance at a global level, the exercise of homogenising *power over* nation states both culturally and economically, that gives continuity to patriarchy as the Western gender structure, hence its coloniality. The social structures that naturalised this global order and the positioning of labour as the principal definer of male identity are essential for understanding violence as a process of coloniality. A political economy of gender analysis therefore implies analysing the role of labour, production, employment and global, national and local cultures and economics of exchange, as these relate to gender (Chant and Craske, 2007; Mies, 1998).

Considerable research has been dedicated to unpacking the process through which Western patriarchal colonialism defined male and female roles, and how a system that originated in Europe came to be transferred to colonies (Connell, 2016; Jaimes Guerrero, 2003; Mies, 1998). For Latin America, this happened at a defining moment for global history and ideology, for ‘Spanish state-making [during the 16th and 17th centuries] was coterminous with the initial processes of European state-making’ (Silverblatt, 2009: ix) and, through colonialism, this European lifestyle was established as *the* universal form for human society. Race and gender as structures were defined as part of this historical process, at a time in which moral, religious, economic and ideological bases of human and societal organisation were renewed and redefined, making the hierarchisation of race and gender inseparable from the economic process of colonialism. Historical evidence suggests that women had public roles in early colonial and pre-colonial times, holding positions both in politics and as important crafts- and tradeswomen (Gamlin, 2020; Kellogg, 2005; Mies, 1998). Feminist historical research largely agrees that colonisers and subsequent post-colonial governments in Mexico, as in other parts of Latin America, saw women minoritised and minimised, confined to the domestic sphere (Orozco, 2019), a process that Cusicanqui (2012) refers to as the ‘colonial seal of the exclusion of women’. Through a process that Jaimes Guerrero (2003) refers to as trickle-down patriarchy, colonisation ‘turned women into a being who can be mistreated, raped, exploited, worked to death’ (Orozco, 2019: 137) and, in so doing, elevated the status of men. Legal documentations evidence how these patterns became law during the 19th and 20th centuries, with marriage giving men the right to kill their wives until 1871, while household and domestic tasks were legally a wife’s responsibility until 1974 (Alonso, 1995). In contrast, historical research and indigenous writing suggest that pre-colonial gender relationships were ‘complementary’, so although these were not fully equal, male and female roles were likely to have been more egalitarian than those established under patriarchy (Celentani, 2015; Kellogg, 2005). These data are important for understanding the connections between colonial structures and a relational gender system where colonised men, themselves experiencing the effects of racialisation, became the colonisers of women, hence while men lost power over their territory, they gained control over women (Jaimes Guerrero, 2003).

Gender-based violence (GBV), a violence that is the result of normative role expectations that are associated with unequal structures of gender (Jaimes Guerrero, 2003; Segato, 2010a; Smith, 2003), is crucial to the coloniality of gender as a process, although it is by no means limited to the coloniality of gender. Mies (1998) sustains that violence was the mechanism through which men gained control over resources, such as food, in the very early days of human civilisation, suggesting a lengthy historicity to the use of force to control women. However, GBV can be against people of any gender, and refers to violence that is the result of the gender expectations and behaviours of aggressor and victim. It is also a relational social process, wherein there are meanings attached to the violence that are about this relationship.

### Stigma and the formation of gender identity

In its dominant presentation, masculinity is an identity that is dependent on men’s productive role, making their sense of masculinity conditional on their ability to perform the aspired after hegemonic gender role (Connell, 2005). Hegemonic masculinity is achieved relationally, and it is this process of identity construction that is interrupted and shaped by stigma. The social context that is the breeding ground of stigma is the same social and spatial location within which gender identity is formed, and these two interact as part of these processes.

Goffman’s use of stigma as an organising concept and ‘a way of seeing, classifying, and understanding a vast array of discriminatory social attitudes and practices’, visibilises its role as a form of governmentality. Strategies of identity management, such as edgework, are used to mitigate the emotional and social impact of stigma – making stigma also a form of social control (Tyler and Slater, 2018: 729). One aspect of this is relational, within a household dynamic; the other refers more directly to gendered productive roles. At a profane level, productivity is reflected in the acquisition of wealth and consumer products that fuel profit and growth. More fundamentally, however, consumption has become a ‘*sine qua non* of social dignity – a passport to personhood’ (Wacquant, 2008: 30, cited in Tyler and Slater, 2018: 734) that intersects with gender identity. Poverty stigma, the curse of being poor in a society or location of wealth – a common experience in Ciudad Juárez that is emphasised by its juxtaposition with El Paso – is a creator of identity, and the stigmatisation of modern poverty intersects with gender identity, with poor men and women acquiring specific negative stereotypical characteristics.

## Violence as a function of globalisation

The interacting processes of gender identity, racialisation, coloniality and violence operate at interpersonal levels and at the macro level, as processes in relation to society (Winton, 2014). Here I will describe their role and functionality in relation to urban development and the production of gendered space in Juárez. Tsing’s (2005) ethnography of global connections usefully locates these processes as essential to the functioning of globalisation, connections that the author describes as ‘friction’. ‘A study of global connections shows the grip of encounter: friction (…) as a metaphorical image, friction reminds us that unequal encounters can lead to new arrangements of culture and power’ (Tsing, 2005: 5). Global forms, such as *supply chains* that exploit cheap labour, *frontiers* between two countries and the categories of *race* and *gender*, are universals produced through colonial encounters. Globalisation institutionalises and naturalises these structures (e.g. gender hierarchies) and their accompanying cultural practices, while urban spaces, such as Juárez and El Paso – and at a wider level nation states – are made through these uneven encounters. Violence is necessary to this ongoing process of building transnational political and cultural ties, as these connections thrive by reinforcing hierarchies, differences or inequalities – gendered, racial, cultural and economic – without which globalisation and exchange would be pointless. Since the agents in these interactions hold different levels of power, they are wrought with violence: normalised exploitation, and structural and interpersonal violences, keep these differences – low wages, gender inequalities, social hierarchies – in motion, while also worlding places.

Not all locations are hubs of global connection, but Juárez is one of these. It is a location where globalisation has brought together different groups of people on unequal terms, stigmatising one and elevating the other on the basis of their relationship with the universals. The ethnographic works of Bourgois (2003) are notable for analysing this dynamic and its resulting social consequences. Bourgois intimately examined the intersecting phenomena of race, class and poverty to explore how marginalised sectors of US society, that might typically dwell in locations of stigma, earn respect and navigate their living circumstances through varying forms of violence. In Latin America, more recent studies have focused on the social acceptability and social role of violence in contemporary contexts or urban marginality (Baird, 2012; Hume, 2004).

There is a dearth of accurate homicide statistics on Mexico due to the very high numbers of missing people; however, by the end of 2018 a rate of 29 homicides per 100,000 people (a total of 35,964 homicides) had been recorded in Mexico, making it the most violent year this century (Secretariado Ejecutivo [Mexican Executive Secretary], 2019). In the first nine months of 2019, there were an average of nearly 100 homicides per day (Phillips, 2019). Also in 2018, Juárez saw 1247 murders, a rate of 88/100,000 (Secretariado Ejecutivo [Mexican Executive Secretary], 2019), and data overwhelmingly suggest that the ratio of male to female homicide, around 9:1, changes little, with figures increasing and decreasing in parallel.

This pattern of male and female mortality is a gendered health effect: masculinity is a risk factor for experiencing a violent death, and is a result of the process through which men and women have been drawn into history and how they live out and define their gender identities. Gendered violence (Hume, 2004) has also become normalised as a central element of gendered and social relations. Male-on-male violence is as much about male gender identity as male-on-female violence, as both forms seek to reinforce the universal of male superiority. As Chant and Craske (2007: 293–294; translation mine) describe:

Changes to the market economy, which have undermined the capacity of men to fulfil their regular duties as ‘family providers’, have incubated a perceptible crisis in ‘masculine identity’ in many places in the world, not only in Latin America.

The links between criminal and interpersonal violence and socio-economic exclusions make this historically generated structural violence (Arteaga Botello and Valdés Figueroa, 2010). Within this process, the extreme forms of exploitation and inequality that came with colonialism – structural violences – have come to be seen as universals within the natural order of society. The idea that varying forms of violence are somehow an inherent aspect of social relations in Latin America has become normalised (Gamlin and Hawkes, 2017; Orozco, 2019). Death and disappearance, which are part of the recent and distant history of most Latin America nations, constitute processes of structural violence embedded in the collective conscious, culture and identities. However, a decolonial perspective on violence reminds us that this was not a native tradition. Colonialism was established through violence, and institutionalised violence was the social process through which hierarchies of race and gender were generated, sustained, challenged or revoked. These social structures did not exist in this form before. Decolonial analyses are beginning to demonstrate how violence was introduced as a management (punishment) strategy for moral and social deviance (Gamlin, 2020), replacing divine punishment. This link, however, requires further investigation.

### Gender and violence in the making of urban space

Research with men in Mexico has shown a clear pattern linking structural and interpersonal violence: as opportunities for men to make a living are restricted, domestic violence increases (González Montes, 2012; Falcón, 2004). A recent analysis of femicides provides evidence that a major factor in this is the collapse of dominant or universal models of femininity and masculinity, through changes to men’s and women’s roles in the labour market impacting on the gendered division of labour (Arteaga Botello and Valdés Figueroa, 2010). The epidemic of femicides in Mexico began in Ciudad Juárez where with the signing of NAFTA, the labour market became increasingly defined by US consumer culture. Demand for goods, from narcotics and pharmaceuticals to clothing, televisions and other household items, generated large-scale manufacturing along the US–Mexico border, drawing in young women from across the country. A location of neoliberal globalisation that both generates and feeds alienation^1^ (Lyng, 1990), Juárez simultaneously produces a loss of identity from non-meaningful and uncreative work and feeds consumer needs to re-create identity through consumption. To use Segato’s (2010b: 70) words, Juárez is ‘an emblematic place of economic globalisation and neoliberalism, with its insatiable hunger for profit’. Tsing (2005: 27–28) refers to such border spaces of accelerated accumulation as ‘resource frontiers’; these are ‘edges where the expansive nature of extraction comes into its own … they confuse the boundaries of law and theft, governance and violence, use and destruction’. The resources to be converted into ‘corporate raw materials that … [had not been] previously commodified’ (Tsing, 2005: 33) were young women. It is simultaneously a location of inequality and marginality, where through the process of capital accumulation, as workers and the unemployed ‘gaze up’ (Tyler and Slater, 2018: 729) to lives and consumer culture in the US, stigma is generated. Hence the importance of Juárez’s border location to the stigma–violence nexus.

The historicity of this patterning is intertwined with Mexico’s industrialisation and subsumption within the global economy. Article 27 of the 1910 post-revolutionary constitution redistributed large private holdings as communal land, in the form of ejidos, that could not be bought or sold, legislation that aimed to guarantee subsistence to peasant farmers. Agricultural production became entwined with gender, a link that was reinforced through popular cultural representations (Gamlin and Hawkes, 2017; Monsiváis, 2013) connecting cultural and gender identity through revolution, land and kinship patterns – but rural life was by no means a peaceful idyll. The revolution of 1910–1921 saw the population decline from 15.2 to 14.3 million (Kemper and Peterson Royce, 1979), and this predominantly rural violence, followed by the *Cristero* (religious) wars, saw migration towards cities, reaching an urbanisation rate of 20% in 1940. It was the huge and dramatic increase that took place largely between 1940 (19.7 million) and 1980 (66.8 million), and beyond, that led to rapid urbanisation (Alba and Potter, 1986). This was also a period of some stability as inward-looking industrialisation funded a strong state and linked employment, health and education infrastructures, providing sustenance for the patriarchal structures of economy and society.

Then, in the wake of the 1980s debt crisis, Mexico took a neoliberal turn, one of the centrepieces of which was reforming Article 27 of the constitution to allow the buying and selling of ejidal land. This un-making of communal structures, together with accelerations of consumer industrial production, began to disorder the structures and practices of gender away from the stable patriarchal patterns established in the era of nation building. Neoliberalism took hold in Mexico at a time when the ‘youth’ (18–25-year-old) population was approaching its demographic peak (Reguillo, 2012), with the largest ever cohort of people in this category in 2005, but infrastructure did not keep pace with population growth. Illustrating this, the term ‘nini’^2^ was coined in this period to refer to the cohort of young people who were not in work or school (Reguillo, 2012). Identified by Ballí (2009) in her description of Ciudad Juárez, these unemployed youngsters were an easy target for employment in the organised crime sector. In what Reguillo refers to as the Pirate Imaginary, this young group came to value extra-legal activities as a means of generating income. There is also a blurring of lines between formal and informal as, gazing up to corporate structures, criminals behaved like businesspeople, demonstrating the masculine traits of competition, rationality and violence, a ‘logic’ that was reproduced in public discourse (Wright, 2011: 719).

Authors of work on Mexico’s femicide epidemic converge on the fractious relationship between globalisation and labour migration and their impact on gender dynamics (Monárrez, 2002, 2010; Orozco, 2019; Segato, 2010a; Wright, 2011). In contrast, male-on-male violence has rarely been theorised as gender-based violence. The violences discussed in this article – both male-on-male violence and violence against women – are entwined with economic restructuring as it impacts on urban and peri-urban spaces. As maquiladoras began to line the northern border, new forms of largely female employment in production line manufacturing coincided with rural decline. This expansion of female employment destabilised the political economy of gender and subsequent social roles upon which Mexican identity was to a great extent built (Monsiváis, 2013). Femicide then comes as a social message or protest, communicating men’s gendered right to exercise power over women (Arteaga Botello and Valdés Figueroa, 2010). Similarly, male-on-male violence can be viewed as a process of gender affirmation, a gender identity forming action (Lyng, 1990; Winton, 2014), confronting the multiple and intersecting disadvantages that are forged through globalisation. Gender-based violence in this context is a stigma-fuelled violence, an action ‘that expects a certain type of recognition, in summary, an action that is compatible with male identity’ (Azaola, 2009: 212; translation mine). It is in this functional role as a process of self-realisation that violence becomes a form of edgework (Lyng, 1990), changing male identity and the spaces that males inhabit.

As an agent of identity, edgework functions to redefine a set of circumstances, giving meaning in a context of urban deprivation and alienation, countering territorial stigma with purposive action. Symbolic violence, defined as ‘violence which is exercised upon a social agent with his or her complicity’ (Bourdieu and Wacquant, 2012: 272), refers to a form of violence that is acted out by and against the victim. To use the authors’ words, ‘social agents [of symbolic violence] are knowing agents, who even when they are subjected to determinisms, contribute to producing the efficacy of that which determines them insofar as they *structure what determines them*’ (Bourdieu and Wacquant, 2012: 272; emphasis mine). Bourdieu used gender domination as the paradigmatic form of this concept, suggesting that symbolic violence occurs as women accept a range of postulates and axioms regarding their domination by men. In so doing, they position themselves for emotional abuse in the form of shame, humiliation and timidity, while accepting the universality of their domination, making this process both gendered and gendering (Bourdieu, 2007). This original version is problematic inasmuch as it depicts women as objects of symbolic violence, seemingly acting without agency, while at the same time blamed for their subordinate status. Here I use the concept to demonstrate the process by which men in contexts of urban marginalisation bring violence upon themselves in the form of social stigma, adaption to their racialised subordination and gendered shame, and how they respond to this by generating other forms of physical violence. Using the concept in this way challenges the idea that there is a loss of agency; on the contrary, as I explore in the following section, it is a symbolic action. Symbolic violence therefore becomes the bridge between ongoing colonial, racial and gendered violences and *edgework*, the process through which male-on-male gender violence redefines disrupted identities as a form of protest masculinity, a response to powerlessness and ‘claim to the gendered position of power, a pressured exaggeration of masculine conventions’ (Connell, 2005: 111).

## Resisting coloniality: From stigma to edgework

The process of spatial ‘relegation’, as Wacquant described the identity impacts of territorial stigma, ‘takes the form of real or imaginary consignment to distinctive psychospatial formations, variously and vaguely referred to as “inner cities”, “ghettoes”, “enclaves”, “no-go areas”, “problem districts”, or simply rough and different spaces’ (Wacquant, 2016: 1077).

In this section, I take a coloniality lens to spatial and racial stigma as these intersect in specific locations, to explore edgework as the process through which coloniality is resisted and experienced. Building on Lyng’s conceptualisation of edgework as both voluntary risk-taking and means of identity management (stigma mitigation), I demonstrate how it ties violence into the ongoing dynamics of coloniality.

### Territorial stigma

The construction of race-based hierarchies occurred in parallel to nation and gender building, under colonialism and the post-colony (Fisher and O’Hara, 2009), with state education systems mobilised to instil cultural homogenisation, including the reduction of native languages and cultures in much of Latin America. As Menéndez (2002: 387) unequivocally states, ‘violence had a central role in the construction of society, and simultaneous rupture with determined [non-European or indigenous] forms of life, […] generating a collective imaginary that accelerated and justified its disappearance’. Race, like gender, is a product of colonialism, with European and white universality (Ahmed, 2007) defined in relation to the other, the non-white. Multiple authors affirm the inseparability of race and gender (Jaimes Guerrero, 2003; Smith, 2003), defining an intersectional biopolitical process through which states exercise control over the behaviour of colonial subjects, and emphasising the dichotomisation of nature/culture, irrational/rational and civilised/savage. At its intersection with gender, this produced a specific type of man and woman, with the hypersexualisation of native women contrasting with the sexually restrained bodies of white women, and savage (violent) native men contrasting with civilised white men (Smith, 2003; Wiesner, 1993). Black, native or dark bodies are both ‘othered’ and degraded. A ‘good’ woman, as Ballí relates in the contexts of Juárez, aspires to replicate the ideal of a white woman, not one of unrestrained sexuality, as the victims of femicide have been characterised. Hence universalised hierarchies of race (racism) provide biopolitical justification for the denigration of non-white people, or, to use Wolfe’s term, ‘the elimination of the native’ (Wolfe, 2006).

Racial inferiority is defined in relation to whiteness, a term that describes the ‘ideal’ identity of a modern capitalist human and infers a set of modern cultural practices (Echeverría, 2016). It is also a location within the world associated with white bodies, articulating how race extends into physical spaces. Certain spaces are considered ‘white’ while others acquire a stigma of not being white. ‘A certain white appearance […] is required to define the ideal identity of the modern capitalist human’ (Echeverría, 2016: 11), making whiteness a cultural identity and a social and bodily orientation. Rural Mexico is associated with indigeneity, non-whiteness, backwardness and underdevelopment, while urban spaces are associated with modern and mestizo (mixed race) contexts. As migrants move urban-wise in search of survival, their residence on the fringes of cities in similarly marginalised contexts brings with it or perpetuates this non-whiteness, reinforcing stereotypes of underdevelopment, irrationality and barbarity.

At its intersection with specific spaces, whiteness creates *territorial stigma*, ‘that most salient symbolic property, that together with fragmentation of wage labour on the material front constitutes advanced marginality’ (Wacquant, 2014: 1271). These are spaces that become an ‘anchor of social discredit’, that are defined by and define colour, pictured as ‘vortexes and vectors of social disintegration’ (Wacquant, 2014: 1274). Race and location are also a cultural identity, and Winton describes how urban gangs foment their own cultural identity as one that embodies marginality, race and location, creating ‘local institutional landscapes’ that respond to ‘structural disadvantages’ (Winton, 2014: 403). The term Protest Masculinity is used to define masculine identities that are a response to the ‘humiliating social consequences’ of emasculation (Tomsen, 1997: 94). The search for honour and respect evidencing itself as ‘local power displays’, collective social processes that are experienced as ‘liberating social activities’ generating group notions of *edgework*, ‘experiences that produce a sense of self-realisation (Lyng, 1990: 860).

### Edgework as resistance

Gang violence generally occurs in contexts of urban marginality where, bereft of opportunities, young men seek new forms of social and cultural identity as well as means of survival (Winton, 2014). Drawing on Marx’s concept of alienation, Lyng (1990: 855) explores how edgework achieves this by ‘negotiating boundaries between chaos and order’, a fitting definition in the border context of Juárez. Edgework defines boundary lines in many ways – life vs death, sanity vs insanity and an ordered sense of self and environment vs a disordered sense of self and environment (Lyng, 1990: 857) – but it also plays to the dichotomies created by globalisation: masculine vs feminine, white vs non-white, developed vs undeveloped as these are constantly negotiated. Specific forms of violence aim to produce meaning in a context of marginalisation where lifestyles, gender, race and location are defined by stigma. In edgework, risky, collective-action ‘voluntary risk taking’ (Lyng, 1990: 852) transforms situations of vulnerability, ‘autopowering’ the subaltern by demonstrating control over disorder and creating powerful identities.

In Latin America, edgework has arisen from coloniality through the making and re-making of gender, race and post-colonial statecraft, or the *coloniality* that transformed social spaces and their inhabitants into stigmatised places and people. Connell (2016: 6) refers to this stigmatisation as the ‘dispossession’ central to colonisation itself, a ‘crucial condition for the formation of gendered work-spaces in colonial society’. In this sense, edgework is the process of forming and reforming gendered and racial identity in the fallout of globalisation, a resistance to their colonial location and its impositions. Although patriarchy and race were established and reinforced within Europe and beyond, largely before the 20th century (Wiesner, 1993), it is the manner in which they were reproduced in the post-colony through globalisation, and later disrupted by new forms of capital accumulation, that was catalytic of edgework.

A decolonial analysis of urban margins shows the mode of production of space and the role of colonial and post-colonial states in this process. As an example, informality as a method of organising life in marginalised suburbs is not an unregulated process, ‘but structured through various forms of extra-legal, social and discursive regulation … producing an uneven geography of spatial value’ (Roy, 2009: 826). A coloniality analysis reveals how decisions about structures and infrastructures and the organisation of space that happen both at the level of policy and of community are not random but give preference to modern (colonial) forms of social organisation, hence the prioritisation of whiteness necessarily opens up spaces for edgework and informality. Water scarcity in Juárez is not a simple fact of geography but the result of US–Mexico power struggles along the border that consistently favour US citizens over Mexicans. A second factor operating is Juárez’s culture of impunity (Vulliamy, 2019). The disaffect that is produced through colonialism/coloniality is encouraged by a lawlessness that lies at the intersect of neoliberal governance – the corporatisation of state and social administration – and acute racially-defined marginalisation. As has been discussed throughout this article, globalisation governs Juárez, expanding the conceptualisation of coloniality from that of ‘a state which continues to live by the practices, structures and legacies of European colonialism’ (Quijano, 2000) to that of a state that is effectively governed by the laws of global and financial corporate power structures. The sliding scale of impunity that enables weaker environmental standards in manufacturing facilitates the entrance of weapons into Mexico and fosters a generalised culture of impunity that also benefits the US economy. In the coloniality of globalisation, security functions principally to protect capital interests, a state system that Arteaga Botello and Valdés Figueroa (2010: 13) define as ‘necropolitical’, for the disposability of swathes of its population. Through the lens of coloniality as urban informality coincides with inequalities of race and violence, between past and ongoing forms of colonialism there emerge ‘zones of exception’ (Roy, 2011: 233), where edgework plays with the lines of order and disorder, creating social and real meaning where it has been lost, resisting relegation by dominant political strata.

## Conclusions: The coloniality of gender violence

Neoliberalism, as the global mode of state and social organisation, is dazzling in many regions of Latin America, especially so in Juárez, a city worlded by its location on the US border. In the opening section of this article, I described what a city that has been made in this way can look like; natural resource colonialism that laid the foundations for poor housing and an industrial economy; spatial distribution that responds to the demands of the global economy; poor infrastructure stigmatising neighbourhoods; and how the frontier with the USA has defined a gendered economic development geared towards illegal, exploitative and/or unregulated trade. Can the coloniality in experiences of cities such as Juárez ‘reconfigure the theoretical heathlands of urban and metropolitan analysis’ (Roy, 2009: 820)?

In earlier times, Juárez was defined by border crossings, as US citizens sought sex workers, cheap alcohol and drugs south of the border. NAFTA changed this by expanding mass production in the city that drew in female migrant workers, thus space was recrafted and gendered by international trade legislation, a modern-day colonialism. This movement of women into the public space through employment generated a relative decline in opportunities for men. Bourgois (2003) links late 20th-century re-structuring of the global economy with a crisis of New York’s working-class patriarchy. His empirical work reinforces the notion that the occupational system is the most important process through which men achieve their status, with an absence of opportunities acting as a blockage to manhood. At the interchange of Global North and South, Juárez demonstrates the veracity and longevity of this pattern, but also how this gendered phenomenon re-shapes and is shaped by urban spaces. I extend the concept of edgework, originally conceived to theorise extreme risk-taking, to explain the role of violence in shaping gender identities, urban spaces and ultimately the local and global political economy. The friction between conflicting social, spatial and economic phenomena – location, race, gender, the labour market – has defined the new youth culture and social order and broken modern gender models. Infrastructural inequalities that are colonial or global (from globalisation) in origin have become vectors for violence. So while Juárez may not reconfigure how we theorise urban spaces, it can offer a critical location from which to re-think the awkward urban encounters of globalisation and the coloniality of development as a destination.

In this article, I have theorised urban gender violence – including gang violence and public violence against women – as a consequence not of poverty or urban marginality, but of its location and function within a globalised world, demonstrating how globalisation, as a suspension of the nation state, repurposes historical and gender divisions to serve the global economy.

By applying a coloniality lens, I argue that globalisation is reconfiguring gender in spatially different ways that continue to be defined along historical lines of race and power. Juárez shatters the linear developmentalist idea that there is a destination location for modern cities, an achievable universal of shared prosperity. Coloniality as an axis of inequality structures and constricts Juárez City’s development. The urban distortions that result from a local economy focused on production for an international consumer market create frictions that have pushed gender inequality out of the household and into the public realm, newly changing urban spaces.

The border location of Juárez makes it a location that speaks to the continuity of North–South (coloniser vs colonised) relationality that the concept of coloniality emphasises: the dynamic political economy of gender, racialised planning and contemplations of place, the role of violence in defining and redefining social orders. Theorising urban spaces globally cannot be done from any one city or nation’s experience, but Juárez’s specific location makes it an ideal demonstration of how critical social theories, such as the coloniality of gender, can expose the historicity and globality of urban processes, illuminate the intersection of space and race and alter our interpretations of violence.

While the border location magnifies inequalities such as these in terms of both intensity and visibility, these exist throughout the Global South in contexts where local political economies have not freed themselves from entanglements with or governance by globalisation.

## Notes

1. Referring to Marx’s concept of Alienation, implying the coming into being through relationships of production and consumption in capitalist society.

2. Nini stands for ‘ni escuela ni trabajo’– neither work nor school.
